# Pregnancy with sigmoid volvulus: A case report with literature review

**DOI:** 10.4274/tjod.32767

**Published:** 2015-06-15

**Authors:** Hansa Dhar, Kamal Ahuja, Eman Alyon Nimre, Shreen Ayesha Khan, Mazin Abdulla Arif, İlham Hamdi

**Affiliations:** 1 Nizwa Hospital, Clinic of Obstetrics and Gynecology, Nizwa, Oman; 2 Nizwa Hospital, Clinic of General Surgery, Nizwa, Oman

**Keywords:** Sigmoid volvulus, bowel obstruction, gangrenous bowel, rectal decompression, laparotomy, endoscopic reduction

## Abstract

Sigmoid volvulus refers to torsion of a segment of the alimentary tract, which often leads to bowel obstruction and ischemic changes. Sigmoid volvulus is an acute surgical emergency because delay in diagnosis and management can cause adverse maternal and fetal complications. Sigmoid volvulus typically presents with acute-on-chronic abdominal distension that may develop slowly over 3-4 days. An early and effective resuscitation with fluid replacement, electrolyte balance correction, prophylactic antibiotics and nasogastric decompression is necessary. The standard goals of treatment are to relieve the obstruction, avoid colonic ischemia, and prevent recurrence either by endoscopic decompression or resection with primary anastomosis. A pregnant woman with sigmoid volvulus at 34 weeks and 1 day of gestation presented to our hospital with abdominal pain, vomiting, and constipation. The patient was jointly surgically managed with laparotomy, cesarean section, and detorsion of the sigmoid volvulus, and was discharged in a healthy state on the 5^th^ postoperative day.

## INTRODUCTION

Sigmoid volvulus arises when the air filled colon twists due to its narrow elongated mesentery and lack of colonic fixation to the retroperitoneum. Sigmoid volvulus is the most common cause of intestinal obstruction accounting for 25-44% of cases^(1)^. Symptoms arise either through bowel obstruction or ischemia. This case is presented to highlight that pregnancy can delay presentation and diagnosis, and nonsurgical management can be equally successful in the 3^rd^ trimester provided there is no evidence of complication like gangrenous bowel or perforation. Endoscopic reduction of a sigmoid volvulus has been reported to be successful in 75-95% of cases^(2)^. Fetal and maternal mortality rates have been noted to be high during pregnancy due to delayed diagnosis^(3)^.

## CASE REPORT

A woman aged 25 years, gravida 2, para 1, presented to the emergency department of our hospital at 34 weeks and 1 day of gestation with intermittent abdominal pain, nausea, and projectile vomiting, which she was having for 2 days, followed by worsening constipation. The patient’s previous delivery was by cesarean section for breech presentation, two years ago. On physical examination, she was afebrile and dehydrated, and her pulse rate and blood pressure were 86 beats per minute and 102/66 mm-Hg, respectively. The patient’s chest and cardiovascular examination was normal, but her abdomen had generalized tenderness and was distended. Bowel sounds were initially sluggish and became absent by the second day of admission. Her vaginal examination was unremarkable with a long uneffaced, closed cervix. Abdominal and pelvic ultrasound did not reveal any free fluid in the abdomen. Appendix appeared normal with no appendicular masses or cysts seen. The liver, gall bladder, spleen, and both kidneys were normal in appearance. The woman refused abdominal radiography in view of her pregnancy. Magnetic resonance imaging (MRI) could not be done due to unavailability of this facility in the hospital. At this point, a clinical diagnosis of bowel obstruction was inferred and a surgical consultation was requested. The rectum was found to be empty on examination with no palpable mass. Surgeons ruled out any evidence of bowel obstruction and recommended to continue symptomatic care and observation, nil oral, with intravenous fluid management.

Routine laboratory examination revealed that her hemoglobin was 12.52 g/dL, white cell count was 6.21 10^3^/ ul (2.4-9.5), and platelets were 209.40 10^9^/L (150-450). Serum urea, creatinine, and serum potassium levels were within normal limits. Other investigations including liver function tests, serum amylase, and blood glucose levels were also found to be normal. Routine urine analysis revealed a white blood cell count of 52/ul with a positive culture growth of mixed bacteria for which she was treated with growth sensitive antibiotics. High vaginal swabs were normal on both gram staining and culture.

**Figure 1:**
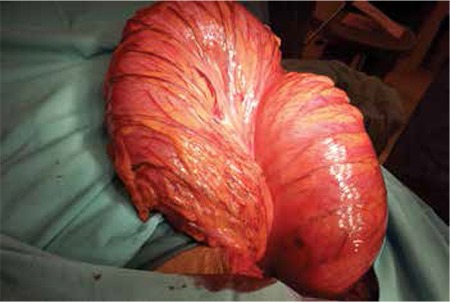
180° anticlockwise twist of a large sigmoid volvulus

**Figure 2:**
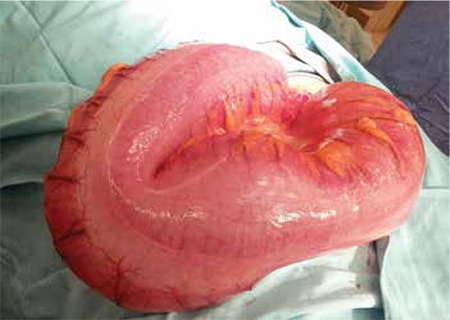
Dilatation of sigmoid colon during cesarean section

The woman stayed in hospital for 4 days and left the hospital against medical advice before a conclusive diagnosis could be made. She later reported to a higher tertiary center where she was seen in the outpatient department and was diagnosed as having a urinary tract infection. Patient then returned back to our hospital and was readmitted by us because there was no relief of her symptoms. A repeat abdominal and pelvic ultrasonography revealed the same findings as the first scan. Surgical team was consulted and a nonspecific clinical diagnosis of intestinal obstruction was made. She was planned for emergency cesarean section with exploratory laparotomy. No effort was made to use endoscopic detorsion in this case because the level and type of obstruction remained uncertain. Cesarean section was performed using a midline vertical incision and a 2000-gram, live, female baby was delivered with Apgar scores 8 at one minute, and 9 at 5 minutes. Sigmoid colon (Figures 1, 2) was found grossly distended with 180˚ anticlockwise twist but absolutely viable with no vascular embarrassment. A non-surgical management of anterograde decompression with detorsion and sigmoidopexy was chosen because her colon was viable and the proximal colon was empty except for gaseous dilation. Bowel resection was not considered to be necessary in this case. Sigmoid colon was detwisted, all air was evacuated towards the rectum and a colo-colopexy and sigmoid fixation was performed with the transverse colon to the lateral pelvic wall. The rest of the peritoneal survey was normal. Finally, an anal dilatation was performed and a rectal tube was inserted. On postoperative assessment, there was resolution of the abdominal distension and passage of flatus. The patient was started on fluids until she could tolerate a full diet. A follow up abdominal X-ray on the third postoperative day showed absent bowel-loop distention or fluid levels and the rectal tube was removed thereafter. Her postoperative period was uneventful and she was discharged on the 5^th^ postoperative day. Follow-up review in the surgical outpatient department was conducted six days later and she was found to have no problems. Her passing motion was normal. To date, there has been no report of recurrence. She has been fully informed about the possibility of recurrence and is being carefully followed-up in the surgical outpatient department.

## DISCUSSION

Volvulus is twisting of a bowel loop on its own mesentry, which causes bowel obstruction, occlusion of venous return, and may lead to gangrene and perforation if it is not quickly relieved^(4)^. Sigmoid volvulus is the most frequent site, followed by cecum, ascending colon, and transverse colon^(5)^. Khan et al. found 84 cases in their latest review of the literature, whereas Ahmed reported fewer than 90 cases^(6,7)^.

Pregnancy increases the incidence of sigmoid volvulus because the enlarging uterus can cause a redundant or abnormally long sigmoid colon to twist. It is very often seen in multiparous women in the 3^rd^ trimester and may sometimes mask the clinical picture because abdominal pain may be a normal finding, as happened in our case^(8)^.

In many series, reluctance for radiologic examination owing to pregnancy is observed^(8)^. A dose of 0.01 Gy is dangerous, with a 1/1000 risk of congenital malformation, whereas a low dose of 0.001 Gy is used in abdominal X-ray. The goal of evaluation is to urgently establish a diagnosis and to rule out other causes of abdominal pain. The other modalities of diagnosis are abdominal computerized tomography scan and MRI, which show characteristic whorls, representing the twist in the mesentry^(9)^. A water soluble enema such as gastrografin may be useful in selecting patients. Sigmoidoscopy or colonoscopy are therapeutic procedures, but may be used to confirm diagnosis. Obstruction of the intestinal lumen and impairment of vascular perfusion occur when the degree of torsion exceeds 180° degree and 360° degree and a variant of sigmoid volvulus (ileosigmoid knotting) occurs when ileum wraps itself around the sigmoid, usually in a clockwise manner^(9,10)^. Other than a redundant sigmoid loop and a narrow base of the mesocolon, none of the possible causes like adhesions, previous pelvic inflammatory disease or gastrointestinal abnormalities were observed intraoperatively in our case. Bowel ischemia, gangrene, and perforation leading to peritonitis are the complications of untreated volvulus, which lead to increased maternal and fetal death^(7)^. A gangrenous bowel should not undergo detorsion but should be resected to avoid systemic dissemination of toxic substance^(9)^. Many series highlight non-colectomy procedures that may be beneficial in individualized cases for management of sigmoid volvulus^(7,8)^. Machado and Machado managed a case of sigmoid volvulus that complicated pregnancy by resection and primary anastomosis and encountered another case of ileosigmoid knotting with gangrenous ileum and sigmoid colon in a male patient who was chronically constipated later in the same year^(9)^.

Bowel obstruction in pregnancy is mainly due to adhesions, volvulus, intussusceptions, hernia, appendicitis, and carcinoma and need surgical intervention, except in uncomplicated sigmoid volvulus, which can be managed non-surgically as in our case, and many other reported cases^(8,10)^. Samarakoon et al. experienced a similar case that resembled ours^(11)^. Their patient had volvulus at 30 weeks of gestation and was managed by midline laparotomy with anterograde decompression and derotation with anchoring of sigmoid mesentery to the lateral pelvic wall. Volvulus is derotated and deflated by sigmoidoscopic placement of a rectal tube to postpone resection till the postpartum period, but studies showed that conservative decompression may be difficult as the large uterus hinders the untwisting of the colon in the last trimester^(10)^. The reported maternal and fetal mortality are 6-20% and 35%, respectively, and Alshawi suggested that the preferred options might be nonoperative colonoscopic detorsion and rectal tube compression in the first trimester, colectomy in the second trimester, and non operative treatment in the third trimester till fetal maturity, followed by sigmoid colectomy after delivery^(11,12)^.

Recurrence of the volvulus is very common, so elective surgery to remove the redundant mobile sigmoid with anastomosis is often performed. The series of Liang et al. reported that laparoscopic elective surgery after successful colonoscopic decompression may be a good choice for a selected group of patients in terms of minimized surgical complications and quick recovery^(13)^. Endoscopic sigmoidopexy has been used instead of surgical resection for patients who are at high risk for surgical complications.

Atamanalp used flexible sigmoidoscopy and then performed definitive surgery to prevent recurrent volvulus^(14)^. Immediate laparotomy is needed if endoscopic detorsion fails or in patients with signs and symptoms suggestive of peritonitis. The presence of bowel gangrene and coagulopathy strongly predicts mortality, which suggests that prompt diagnosis and management are essential. The entire bowel should be examined for other areas of obstruction. Bowel viability should be carefully assessed as segmental resection with or without anastomosis may be mandatory in some cases^(1)^.

## CONCLUSION

Although it is not a common cause of bowel obstruction, sigmoid volvulus needs early diagnosis and treatment to avoid serious maternal and fetal complications. Urgent surgery in bowel obstruction in pregnant women is mandatory. Non-surgical management can have a successful outcome provided uncomplicated cases are selected. Resection with primary anastomosis is the gold standard management in the presence of gangrene and perforation. Awareness among clinicians needs to be increased to avoid catastrophic events in such cases.
